# Effectiveness of smartphone technology for detection of paediatric ocular diseases—a systematic review

**DOI:** 10.1186/s12886-025-04160-2

**Published:** 2025-05-30

**Authors:** Sruthi Vijendran, Yash Alok, Neetha I. R. Kuzhuppilly, Jayasheela R. Bhat, Yogish S. Kamath

**Affiliations:** 1 Department of Ophthalmology, Kasturba Medical College, Manipal, Manipal Academy of Higher Education, Manipal, Karnataka 576104 India; 2 Department of Community Medicine, Kasturba Medical College, Manipal, Manipal Academy of Higher Education, Manipal, Karnataka 576104 India; 3 Consultant Optometrist, Karthik Nethralaya Institute of Ophthalmology Pvt Ltd, Bangalore, 560050 India

**Keywords:** Smartphone, Mobile applications, Eye diseases, Visual disorders, Paediatric, Child

## Abstract

**Background:**

Artificial intelligence has become part of healthcare with a multitude of applications being customized to roles required in clinical practice. There has been an expanding growth and development of computer technology with increasing appearance in the ophthalmological universe with roles in detection of most ophthalmic diseases. This article attempts to study the efficacy of smartphones and their applications in detection of paediatric eye diseases.

**Methods:**

On 24 January 2024, a comprehensive search was performed across five databases—PubMed, Scopus, Web of Science, Cumulative Index to Nursing and Allied Health Literature, and ProQuest—focusing on studies assessing smartphone-based disease detection and diagnostic accuracy compared to validated methods. Keywords and MeSH terms, including "smartphone," "eye diseases," and "children," were combined using Boolean operators and eligible studies were obtained. The inclusion criteria covered studies from 2000 to 2023, involving children under 18 years, and reporting diagnostic outcomes. Exclusions included studies not exclusive to eye disease, purely adult population studies, reviews, studies with non-availability of full text, and studies exploring other uses of smartphone and designs lacking diagnostic efficacy analysis. Article quality was assessed using the Joanna Briggs Institute Critical Appraisal Checklist.

**Results:**

A total of 2054 articles were retrieved. After removing 1112 duplicates, 507 records were excluded through title screening, followed by 333 through abstract screening. A full-text review of 83 articles led to the inclusion of 33 studies, involving 16,015 participants. Most of the studies (28, 84.84%) were of high quality, with five (15.15%) of moderate quality. Twelve smartphone applications assessed refractive errors using visual acuity tests or photorefraction, five detected amblyogenic risk factors, six identified strabismus, and three targeted leukocoria. Additional applications evaluated stereoacuity (two), eyelid position (one), chalazion (one), corneal diameter (one), and retinopathy of prematurity (two). Overall, these applications demonstrated the potential of smartphones in paediatric eye disease detection.

**Conclusion:**

Smartphone applications are effective tools for detecting important causes of childhood eye disorders such as strabismus, retinopathy of prematurity, chalazion, and refractive errors. These technologies offer promising opportunities for teleophthalmology and integration into routine clinical practice.

## Background

With the ongoing advancement of computing technology, massive data collection, and imaging techniques, artificial intelligence applications have permeated daily life and have several applications in the healthcare industry, especially in ophthalmology which relies heavily on imaging technology [1].

In healthcare its use in various specialities is varied including its use in medical imaging and diagnosis, calculation of drug dosages, and dates. It is also used in health records maintenance, patient data archiving and disease detection and risk factor stratification. Outbreak prevention, drug development and customization, patient education, counselling, self-surveillance, telemedicine are few of the myriads of applications. Various applications of artificial intelligence have been found in Alzheimer’s, brain disorders, cancer, cardiovascular disease, diabetes and stroke [1, 2].

In Ophthalmology, smartphones and deep learning methods have aided in detection of refractive error, to retinal pathology including retinopathy of prematurity [3–35]. Being a common household device, the user-friendly platforms make it socially acceptable among adults and children alike [36].

Globally, 2.2 billion people are visually impaired due to either distance or near visual impairment and nearly half of it can be attributed to treatable causes such as refractive error [37]. If not detected early it can lead to ocular complications such as amblyopia, strabismus and retinal disorders. Most amblyogenic risk factors(ARF) have better prognosis if detected early in childhood [38].

There arises a need for innovative portable solutions such as smartphone which aids in tackling the issue of untreated refractive error, including other common paediatric diseases. In ophthalmology, smartphones have been documented in disease diagnostics, prognostication, and supporting in treatment and compliance of disease management such as in cataract and amblyopia [39]. There are also roles of smartphone technology in measuring compliance to wearing glasses, low vision devices and audio-visual aids for visually impaired [40]. By presenting a game-like interactive and innovative platform, it can facilitate the evaluation of children by capturing their attention and cooperation for preliminary tests of screening and in communicating the condition easily to the care providers such as parents and healthcare workers, for decision making and remote practice of medicine [41]. Currently there are a multitude of applications that exist with the purpose of aiding clinicians in various ophthalmological roles such as diagnostics and treatment [42]. Hence it is essential to study the types of applications available in smartphone in terms of reliability and its application in the study of paediatric eye disease.

Conventional methods though the golden standard for detecting eye disorders, are often less portable to deploy in community settings [43]. In this systematic review a critical analysis of efficacy of smartphone applications in the detection of paediatric eye diseases with potential to shift the trends of eye health care delivery will be made.

## Methods

### Data sources and search strategy

The review process followed the Prisma-P 2015 guidelines for conducting the study [44]. The PRISMA 2020 Abstract checklist and PRISMA 2020 checklist were used as guidelines to report the review [45]. As available literature was used to report the data, approval from institutional ethical committee and informed consents of patients were not needed.

The search strategy was developed by a reviewer trained in evidence synthesis (S.V.). This was validated by another reviewer (Y.A). We conducted a search on 24 January 2024 in databases of PubMed (MEDLINE), Scopus (Elsevier), Web of Science (Clarivate Analytics), CINAHL complete (EBSCO) and ProQuest for articles reporting on disease detection, diagnostic accuracy, describing the technique of detection through smartphone, with comparison to a valid and reliable standard. Eligible studies that reported the efficacy of smartphone in the detection of paediatric eye diseases were found using key words and medical subject heading terms for “smartphone”, “eye diseases” and “children” with filters for “English” language. Studies in English, published from year 2000 till 31 st December 2023, were included due to time sensitivity. The link to the search strategy has been listed in Table 1 (added at the end of the document).

**Table 1:** Search strategy for databases searched on 24th January 2024

S.no	Search database	Search strategy	Items retrieved
1	PubMed (MEDLINE)	((((((Smartphone* OR"Smartphone app"OR"Smart phone*"OR"Smartphone application*"OR"Mobile application*"OR"Mobile apps"OR"Mobile app*"OR mHealth OR Android OR App OR IOS OR i-phone OR Phone)) OR ((Smartphone* OR"Smartphone app"OR "Smart phone*"OR"Smartphone application*"OR"Mobile application*"OR"Mobile apps"OR"Mobile app*"OR mHealth OR Android OR App OR IOS OR i-phone OR Phone)[MeSH Terms])) OR ((Smartphone* OR"Smartphone app"OR"Smart phone*"OR"Smartphone application*"OR"Mobile application*"OR"Mobile apps"OR"Mobile app*"OR mHealth OR Android OR App OR IOS OR i-phone OR Phone)[MeSH Subheading])) OR ((Smartphone* [Title/Abstract] OR"Smartphone app"[Title/Abstract] OR"Smartphone*"[Title/Abstract] OR"Smartphone application*"[Title/Abstract] OR"Mobile application*"[Title/Abstract] OR"Mobile apps"[Title/Abstract] OR"Mobile app*"[Title/Abstract] OR mHealth[Title/Abstract] OR Android[Title/Abstract] OR App[Title/Abstract] OR IOS[Title/Abstract] OR i-phone[Title/Abstract] OR Phone[Title/Abstract])) AND (english[Filter])) AND ((((("Eye disease*"OR Ocular OR"Ocular disease*"OR"Vision disorder*"OR"Visual impairment"OR"Vision,low"OR"Uncorrected refractive error*"OR"Refractive error*"OR Amblyopia OR Strabismus OR Myopia OR Hypermetropia OR Hyperopia OR Astigmatism OR"Congenital eye disease*"OR"Childhood blindness"OR"Avoidable blindness"OR Ophthalmology OR"Congenital eye disorder*"OR"Congenital blindness")) OR (("Eye disease*"OR Ocular OR"Ocular disease*"OR"Vision disorder*"OR"Visual impairment"OR"Vision, low"OR"Uncorrected refractive error*"OR"Refractive error*"OR Amblyopia OR Strabismus OR Myopia OR Hypermetropia OR Hyperopia OR Astigmatism OR"Congenital eye disease*"OR"Childhood blindness"OR"Avoidable blindness"OR Ophthalmology OR"Congenital eye disorder*"OR"Congenital blindness")[MeSH Terms])) OR (("Eye disease*"OR Ocular OR"Ocular disease*"OR"Vision disorder*"OR"Visual impairment"OR"Vision, low"OR"Uncorrected refractive error*"OR"Refractive error*"OR Amblyopia OR Strabismus OR Myopia OR Hypermetropia OR Hyperopia OR Astigmatism OR"Congenital eye disease*"OR"Childhood blindness"OR"Avoidable blindness"OR Ophthalmology OR"Congenital eye disorder*"OR"Congenital blindness")[MeSH Subheading])) OR (("Eye disease*"[Title/Abstract] OR Ocular[Title/Abstract] OR"Ocular disease*"[Title/Abstract] OR"Vision disorder*"[Title/Abstract] OR"Visual impairment"[Title/Abstract] OR"Vision, low"[Title/Abstract] OR"Uncorrected refractive error*"[Title/Abstract] OR"Refractive error*"[Title/Abstract] OR Amblyopia[Title/Abstract] OR Strabismus[Title/Abstract] OR Myopia[Title/Abstract] OR Hypermetropia[Title/Abstract] OR Hyperopia[Title/Abstract] OR Astigmatism[Title/Abstract] OR"Congenital eye disease*"[Title/Abstract] OR"Childhood blindness"[Title/Abstract] OR"Avoidable blindness"[Title/Abstract] OR Ophthalmology[Title/Abstract] OR"Congenital eye disorder*"[Title/Abstract] OR"Congenital blindness"[Title/Abstract])) AND(english[Filter]))) AND (((((Child* OR Infant* OR Pediatric OR Paediatric OR Neonate OR Newborn OR Toddler OR Adolescent)) OR ((Child* OR Infant* OR Pediatric OR Paediatric OR Neonate OR Newborn OR Toddler OR Adolescent)[MeSH Terms])) OR ((Child* OR Infant* OR Pediatric OR Paediatric OR Neonate OR Newborn OR Toddler OR Adolescent)[MeSH Subheading])) OR ((Child*[Title/Abstract] OR Infant*[Title/Abstract] OR Pediatric[Title/Abstract] OR Paediatric[Title/Abstract] OR Neonate[Title/Abstract] OR Newborn[Title/Abstract] OR Toddler[Title/Abstract] OR Adolescent[Title/Abstract])) AND (english[Filter]))	736
		**Filters:** English, Child: birth-18 years, from 2000 −2024	
2	Scopus (Elsevier)	(TITLE-ABS-KEY ((smartphone* OR"Smartphone app"OR"Smart phone*"OR"Smartphone application*"OR"Mobile application*"OR"Mobile apps"OR"Mobile app*"OR mhealth OR android OR app OR ios OR i-phone OR phone)) AND TITLE-ABS-KEY (("Eye disease*"OR ocular OR"Ocular disease*"OR"Vision disorder*"OR"Visual impairment"OR"Vision, low"OR"Uncorrected refractive error*"OR"Refractive error*"OR amblyopia OR strabismus OR myopia OR hypermetropia OR hyperopia OR astigmatism OR"Congenital eye disease*"OR"Childhood blindness"OR"Avoidable blindness"OR ophthalmology OR"Congenital eye disorder*"OR"Congenital blindness")) AND TITLE-ABS-KEY ((child* OR infant* OR pediatric OR paediatric OR neonate OR newborn OR toddler OR adolescent))) AND PUBYEAR > 1999 AND PUBYEAR < 2025 AND (LIMIT-TO (LANGUAGE,"English"))	542
		**Filters**: English, 2000–2024	
3	Web of Science (Clarivate Analytics)	(((ALL = ((Smartphone* OR “Smartphone app” OR “Smart phone*” OR “Smartphone application*” OR “Mobile application*” OR “Mobile apps” OR “Mobile app*” OR mHealth OR Android OR App OR IOS OR i-phone OR Phone))) AND ALL = (("Eye disease*"OR Ocular OR"Ocular disease*"OR “Vision disorder*” OR “Visual impairment” OR “Vision, low” OR “Uncorrected refractive error*” OR “Refractive error*” OR Amblyopia OR Strabismus OR Myopia OR Hypermetropia OR Hyperopia OR Astigmatism OR “Congenital eye disease*” OR “Childhood blindness” OR “Avoidable blindness” OR Ophthalmology OR “Congenital eye disorder*” OR “Congenital blindness”)))) AND ALL = ((Child* OR Infant* OR Pediatric OR Paediatric OR Neonate OR Newborn OR Toddler OR Adolescent))	333
4	ProQuest	noft((Smartphone* OR"Smartphone app"OR"Smart phone*"OR"Smartphone application*"OR"Mobile application*"OR"Mobile apps"OR"Mobile app*"OR mHealth OR Android OR App OR IOS OR i-phone OR Phone)) AND noft(("Eye disease*"OR Ocular OR"Ocular disease*"OR"Vision disorder*"OR"Visual impairment"OR"Vision, low"OR"Uncorrected refractive error*"OR"Refractive error*"OR Amblyopia OR Strabismus OR Myopia OR Hypermetropia OR Hyperopia OR Astigmatism OR"Congenital eye disease*"OR"Childhood blindness"OR"Avoidable blindness"OR Ophthalmology OR"Congenital eye disorder*"OR"Congenital blindness")) AND noft((Child* OR Infant* OR Pediatric OR Paediatric OR Neonate OR Newborn OR Toddler OR Adolescent))Limits applied	333
		**Filters**: English, 2000–2024	
5	CINAHL complete (EBSCO)	((Smartphone* OR “Smartphone app” OR “Smart phone*” OR “Smartphone application*” OR “Mobile application*” OR “Mobile apps” OR “Mobile app*” OR mHealth OR Android OR App OR IOS OR i-phone OR Phone)) AND (("Eye disease*"OR Ocular OR"Ocular disease*"OR “Vision disorder*” OR “Visual impairment” OR “Vision, low” OR “Uncorrected refractive error*” OR “Refractive error*” OR Amblyopia OR Strabismus OR Myopia OR Hypermetropia OR Hyperopia OR Astigmatism OR “Congenital eye disease*” OR “Childhood blindness” OR “Avoidable blindness” OR Ophthalmology OR “Congenital eye disorder*” OR “Congenital blindness”)) AND ((Child* OR Infant* OR Pediatric OR Paediatric OR Neonate OR Newborn OR Toddler OR Adolescent))	110
		**Filters**:English, 2000–2024	

The [*] symbol in the above table is a truncation tool for search strategy, used to broaden searches by including various combinations of word endings or spellings

### Inclusion and exclusion criteria

Articles and study designs where smartphone technology was used to detect eye diseases in children (less than 18 years) were included. Articles not exclusive to eye diseases, including only the population above 18 years, reviews and study designs not reporting on diagnostic efficacy of detecting eye diseases with inherent properties of smartphone were excluded. Non availability of full text, and articles exploring other uses of smartphone in ophthalmology were excluded. The inclusion and exclusion criteria have been further elaborated in Table 2.

**Table 2:** Inclusion and exclusion criteria

S.no	Inclusion criteria	Exclusion criteria
1	Cross sectional, observational, prospective, randomized control trials, experimental and retrospective studies were included	Those articles that did not mention eye diseases or visual impairments and outside the inclusion criteria were excluded, articles in which diseases occurring in predominantly older age group above 18 years were studied
2	Research from all countries was included	Technical notes, viewpoint, reviews, case study, case series, protocols, conference proceedings, letter to editor, animal studies, meeting abstracts and those with non-availability of full texts were excluded
3	In this study, only original articles from peer reviewed journals in English language were included	Web based applications, virtual reality programmed in a smartphone, tablet devices used to administer the test were excluded, questionnaire based and not inherently detecting eye diseases, multiple apps in one app, device attachments, or if the application predicts severity not detection of eye diseases
4	Articles based on smartphone screening techniques for detection of eye diseases in children were incorporated	Usage of smartphone for manufacturing devices for aiding low vision individuals for facilitating Braille and other visual aids
5		Usage of smartphone for providing treatment and rehabilitation for diseases such as amblyopia, providing medical education on eye diseases and prevention, to encourage patient compliance for following treatment and follow up visits for diseases such as paediatric cataract, amblyopia, and those used to aid in surgical procedures were excluded
6		Ocular nystagmus detecting applications that leaned more towards the vestibular and neurological pathology than ocular disease, systemic diseases

### Data extraction

Rayyan free software [46] was used to compile the search results. Zotero was used as citation manager. Three reviewers (S.V, J.B, Y.A.) independently screened the review articles. The title and abstract screening were performed in a blinded manner by three reviewers (S.V., J.B, Y.A.). They also assessed the eligibility of the texts among the articles. Inter-reviewer disagreements about the studies were solved by a fourth independent reviewer (N.K) following discussion.

Two independent reviewers (S.V. and Y.A.) extracted the data and entered the data into tables in Word for Microsoft 365 MS-Office (Version 2402, Build 16.0.17328.20124)64-bit using an extraction template.

Data was sought with result classified based on specific groups of eye disorders. Data regarding general demographics and study data (title of the study, first author name, year of study, journal name, study description, methodology and comparators, sample size, and findings relevant to the research question with emphasis on target disease(s) studied with disease prevalence, diagnostic accuracy of the tests applied using smartphone with their conclusions and smartphone related information such as name of the application has been mentioned in Table 3 (added at the end of the document). This tabulated form was constructed using the above parameters with respect to the efficacy of smartphone applications for a group of diseases affecting parts of the eye.

**Table 3:** Data synthesis of articles in systematic review

	**Visual acuity-based applications (12)**							
**S No**	**Title**	**Author, Year, Journal**	**Study design**	**Methodology**	**Sample size**	**Inclusion criteria**	**Exclusion criteria**	**Findings**
1	Visual acuity assessment and vision screening using a Novel Smartphone Application [35]	Zhao et al. 2019, Journal of Paediatrics	Prospective cross sectional (diagnostic test accuracy)	**Peek Acuity** app was downloaded as a free beta from Google Play Store. From 2 m with the observer facing the participant, the test was administered. The child had to signal a swiping motion according to the direction of the optotype “E” and the smartphone was moved progressively closer with vibrations being signalled. This was followed by standard visual acuity tests in random order	111 children	Children between 3–17 years of all gender coming to paediatric eye clinic who could follow instructions	Those that left before evaluation by ophthalmologist	Fifty- four participants had a referable ocular disease, 51 participants had decreased vision, and 22 individuals had strabismus In detecting referable ocular disease- 69% specificity and 80% sensitivity was present. The intraclass correlation coefficient for the first eye and second eye was 0.88[95% CI 0.83- 0.92] and 0.85 [95% CI: 0.78–0.89] respectively
2	Validity and Reliability of Vis-Screen Application: A Smartphone-based Distance Vision Testing for visual impairment and blindness vision screening [33]	Rehman et al., 2023, Medicina	Cross sectional study	At 3 m and 1.5 m, the smartphone with the downloaded **Vis-Screen app** was tested among participants compared to electronic Snellen chart 6 m away with the smartphone held perpendicularly to achieve optimum viewing angle. The letter “E” was portrayed, and swiping was done depending on the direction of the “E” to record visual acuity response	408 participants	Those aged 4 years to 91 years and older, physically fit, good communication skills with reliable mental status understanding the pre-test instructions and demonstration	Those nonconsenting, those requiring emergency care or serious care were excluded	The total number of normal participants was 322. Respectively, 24, 45, and 8 Eyes had Mild, moderate and severe visual impairment. Blindness was observed in 9 of the participants. High sensitivity of 88.4% and 85.4% at 6/12 Line for presenting and corrected visual acuity of right eye was observed. The PPV was observed to be between. 57.9% to 81.7%. The NPV was between 96.8% to 99%. The Positive likelihood ratio and a negative likelihood ratio was between 16.73–73.89 and 0.122–0.45 respectively. For the Snellen chart and the application’s reliability. The kappa value was 0.61. Very high values of intra user PVR and CVR Kappa were obtained (0.85 and 0.79 respectively). The value of intra rater Kappa for PVR and CVR were 0.72 and 0.67 respectively. The Kappa values for agreement between the application and Snellen chart were 0.61 and 0.52 for PVR and CVR
3	Early detection of visual impairment in young children using a smartphone-based deep learning system [11]	Chen et al., 2023, Nature Medicine	prospective, multicenter, observational study,	**Apollo Infant Sight application** presented a cartoon-like stimuli to the children. Deep learning model training was done from 25,972,800 video frames of 3652 children. Phenotypic features of face and ocular movements were analyzed for severity and causes of visual impairment. Comparison was done against test and ambient interference noises for model training	3652 children (≤ 48 months)	Chinese children aged 48 months (about 4 years)	Those with brain illness or mental illness were excluded. The dataset used for module development was excluded from the diagnostic data set	Sixteen common eye disorders were identified, Area under curve (AUC) of 0.859 was observed for the internal data sets compared to external ones. Sixty-seven (22.5%) children had mild visual impairment, and 43(14.4%) children had severe impairment. Based on video clip predicted probability to detect **visual impairment**, For internal validation data sets, Area-under-curve (AUC) of 0.940[95% CI (0.92–0.959)], accuracy of 86.5% [95% CI (83.4–89.0)], sensitivity of 84.1% [95% CI (80.2%−87.4%)], and specificity of 91.9% [95% CI (86.9–95.1%)] was obtained. For external validation datasets, Aan area under the curve of 0.843[95% CI (0.794–0.893)], accuracy of 82.6% [95% CI (77.8–86.4)], sensitivity of 80.9% [95% CI (72.6%−87.2%)], and specificity of 91.9% [95% CI (77.6–88.1%)] was observed
4	Parents'performance using the AAPOS Vision Screening App to test visual acuity in Malaysian preschoolers [19]	Azis et al.,2019, *J*.Pediatr. Ophthalmol	Cross- sectional study	**(AAPOS)**Vision Screening App (application) was set at 3 m and picture based optotypes were used for testing The results of the doctor and parents’ usage of this app were compared to retro-illuminated Lea symbol test visual acuity tests done by optometrist at 3 m distance. VA worse than log 0.26 were referred for further evaluation	195 preschoolers using convenience sampling	Children who came to Selayang Hospital (patients and general population) between 5 to 6 years, December 2017—March 2018; visual acuity of minimum logMAR 0.6	Uncooperative, irritable or attention deficit disorders children, or failure to read the binocular pretest of log MAR 0.6 were excluded	Comparing the application to the traditional Lea symbol chart testing, the results showed 86.6% sensitivity and 78.9% specificity for the right eye visual acuity test, and 79.5% sensitivity and 71.8% specificity for the left eye visual acuity screening The intraclass correlation coefficient between parents and optometrists was good (*p* < 0.001). Overall Cronbach's alpha was more than 0.7
5	Development and Validation of a Smartphone-Based Visual Acuity Test (Vision at Home) [10]	Han et al., 2019, *Transl. Vis. Sci. Technol*	Cross sectional	**V@home** test applying tumbling E optotypes of ETRDS chart was compared with traditional ETDRS chart reading at 2 m (binocular and monocular) for distance testing and 40 cm for binocular near visual acuity testing	113 participants between 13–91 years	Those whose legal guardians provided consent (if below 18 years of age) and those providing consent	No exclusion criteria	The V@home application and ETDRS distant visual acuity showed a mean difference of 0.01 to 0.1 logMAR. The median VA ETDRS chart for the right and left eyes, respectively, in the teenage Chinese group was 0.8 and 0.7. The median was 0.2(0–1.0) logMAR near VA ETDRS. There was a mean difference of 0.01 between V@home and ETDRS. When testing distance VA in the right eye, a Tolerant Quadratic Weighted Kappa (TQWK) of 0.950 was observed. For the ETDRS (0.004) and V@home (0.002), the mean test–retest difference and 95% Limits of Agreement (LOA) were comparable. When evaluating binocular near VA and distant VA in the left eye, V@home and ETDRS agreed similarly The ETDRS revealed that the Australian group's median distance VA in both the left and right eyes was 0.1 and 0.1 logMAR. According to the ETDRS, the median near VA was 0.1(0.0–0.6) logMAR. For both the left and right eyes, the Australian group's levels of agreement were much higher than ETDRS (TQWK 0.742 and TQWK 0.805, respectively). TRRs were −0.003 (95% LOA: −0.129 to 0.123) and −0.016 (95% LOA: −0.289 to 0.257) logMAR for ETDRS and V@home, respectively. TQWK measured 0.812 and 0.968. With a TQWK of 0.736 and a mean difference of −0.068 (95% LOA:−0.098 to −0.038), the application demonstrated good agreement for near VA
6	“Smart Optometry” phone-based application as a visual acuity testing tool among paediatric population [26]	Raffa et al., 2022, Saudi Med J	non-interventional randomized cross-sectional study	“**Smart Optometry**” offers 15 different standardized tests including color vision, contrast sensitivity, Amsler grids including others. Tumbling E optotypes at distance and near visual acuity for smartphone were compared with ClearChart®2 electronic distance visual acuity chart at 6 m and 40 cm Rosembaum pocket vision screener near visual acuity tests	100 children	Paediatric patients aged 5–16 years in 2021	Patients with significant verbal or developmental delay and those with VA worse than 20/200	Median logMAR on conventional distance visual acuity and app measured visual acuity is 0. Mean difference of 0.018 was found for application measured near visual acuity in comparison to standard near visual acuity charts (0.12 ± 0.2). Bland Altman plots did not demonstrate any proportional bias between performance of parents while using the app and clinician or near testing. The app had a sensitivity of 89.3% and specificity of 69.4% in detecting subnormal VA. Sensitivity and specificity in detecting amblyopic risk factors was found to be 58.3% and 83%. The interclass correlation of application-measured VA scores by the caregivers and the clinician were 0.77 using single measures and 0.87 using average measures
7	Reliability of Smart Phone Photographs for School Eye Screening [24]	Srivastava et al.,2022, Children (Basel)	Cross sectional observational	Smartphone photography Visual assessments were done in random sequence. Snellen E chart test at 6 m was used for vision test. Smartphone photo was taken at 1 m with default flash on, subject looking at a target 6 m away. This was followed by distant direct ophthalmoscopy, torch light evaluation and undilated retinoscopy	2520 children	Children from 7–15 years of age, between 2020–2022 were recruited	Those who were uncooperative for the visual assessment or unclear photographs were excluded	The prevalence of ocular morbidity was 10.61% in their study, with 188(7.4%) children being diagnosed with myopia and hypermetropia was found in 41(1.6%) children Joint probability of agreement of 86.9% was found for photographs by observers When smartphone photos were compared to conventional screening techniques sensitivity was 94.69% and specificity was 98.85%, positive predictive value was 90.67% and negative predictive value was 99.37%. The sensitivity and specificity for traditional methods of screening were 81.88% and 97.35% The sensitivity for inter observer recognition of normal and abnormal eyes was 81.67% and 95.41% and specificity was 86% and 99% conventional visual acuity screening. For photographs it was 94.69% and 98.85%
8	Development and validation of a new vision screening test algorithm for public use mobile application- A pilot study [9]	Siti et al.,2018, Med. J. Malays	Cross sectional study	Standard Snellen chart at 6 m and compared with the app showing one optotype “E” at 1.5 m and 3 m presented in 1 of 4 directions	279 participants aged 8- 79 years	Participants who could communicate effectively, had stable mental status, and were physically capable of completing the vision test	Minors without guardians and individuals requiring emergency care were excluded from the study	161 participants with abnormal values were included for the validity study High sensitivity of detecting visual impairment lower than 6/12 with 92.7% and 86.7% for right eye and left eye respectively. Specificity was greater than 89% (89.3%- 99.4%) for all visual impairment levels. PPV for left eye was 0 to 86.8% and right eye was from 50 to 88%. NPV for RE was 86–95.7%, Left eye NPV for all vision levels of app was greater than 90%. Inter observer agreement was satisfactory with Krippendorff alpha values for right and left eye at 0.87 and 0.83
9	Deep Learning–Based Prediction of Refractive Error Using Photorefraction Images Captured by a Smartphone: Model Development and Validation Study [7]	Chun et al., 2020, JMIR Med Inform	Development and validity (CS)	Smartphone built in flash system was used to obtain eccentric photorefractive images at 1 m and its interpretation was compared with cycloplegic refraction values	305 photorefraction images from 164 subjects	Children aged 6 months- 8 years who visited the outpatient clinic Samsung medical center Seoul, Korea for routine ocular examination	Congenital cataract, corneal opacity on central visual axis, visual pathway or extraocular muscle disorders , prior ocular surgery, inadequate cycloplegia and uncooperativeness	221 photorefraction images had refractive error determined by cycloplegic refraction The deep learning algorithm achieved an overall accuracy of 81.6%. The class-specific accuracies were as follows: 80.0% for ≤ −5.0 D, 77.8% for > −5.0 D to ≤ −3.0 D, 82.0% for > −3.0 D to ≤ −0.5 D, 83.3% for > −0.5 D to < + 0.5 D, 82.8% for ≥ + 0.5 D to < + 3.0 D, 79.3% for ≥ + 3.0 D to < + 5.0 D, and 75.0% for ≥ + 5.0 D
10	A One-Step, Streamlined Children’s Vision Screening Solution Based on Smartphone Imaging for Resource-Limited Areas: Design and Preliminary Field Evaluation [4]	Ma et al.,2020, JMIR Mhealth Uhealth	Cross sectional	Measurements included personal information, interpupillary distance, visual axis, refractive error ranges, and strabismus risk and they were analysed with image processing with smartphone at fixed distance compared with the ground truths after evaluation for presence of myopia, strabismus and anisometropia	100 children	Between September 1 and October 15, 100 students between 8 to 10 years were recruited from school infirmaries of Luoyang, Henan	Not mentioned (disorders other than Refractive Error, and strabismus)	16 Strabismus cases were diagnosed, 90 myopia, 4 anisometropia cases were detected The detection sensitivity and specificity were 0.80 and 0.98 for strabismus, 0.83 and 1.00 for myopia, and 0.80 and 1.00 for anisometropia. The accuracies of strabismus detection were 94%, 91% for myopia detection and 99% for anisometropia detection
11	Preliminary Evaluation of a Smartphone App for Refractive Error Measurement [22]	Luo et al.,2022, *Transl. Vis. Sci. Techno*	Cross sectional	Autorefraction, subjective refraction and non-cycloplegic retinoscopy was done. The smartphone app was used at 2 m to detect refractive error first and gradually brought closer if they could not read initially	113 participants between 7–35 years	Participants aged 6 years and older with myopia in at least one eye, astigmatism not exceeding −1.75 D, and no known ocular conditions beyond refractive errors	Eyes with astigmatism of − 1.75 D or more, other ocular disorders like cataract, macular degeneration, and glaucoma	201 eyes of 113 myopes were analysed The two measurements showed a strong correlation (Pearson R = 0.91, *p* < 0.001). Repeatability testing revealed a variation of 0.31 D, with 95% limits of agreement at ± 0.61 D
12	Towards Automating Retinoscopy for Refractive Error Diagnosis [30]	Aggarwal et al., 2022, arXiv- Computer Science	Clinical trial	Patient focused on a LogMAR chart 3 m away. The video mode of smartphone was used and light reflex movements obtained were observed on retinoscopy to arrive at a net estimated refractive power which was compared with subjective refraction including autorefraction and retinoscopy values	128 subjects	Participants aged 7–58 years with consent	Patients with cataracts or active ocular infection/acute eye trauma Unfocussed videos, patients blinking continuously, small pupil	89(48.11%) eyes had refractive error sensitivity of 91.0% and specificity of 74.0% was obtained for the classification of refractive error Low sensitivity and specificity (60%) were found for hyperopia due to low sample size (15). High sensitivity and specificity (80%) were found for myopia. Bland–Altman plot revealed the limits of agreement (95%) as −1.26D to 2.26D. It also showed good agreement measures with mean difference of 0.5 ± 0.9D. Good agreement was achieved with Pearson correlation coefficient r (185) with 0.9 p < 0.01. 43.2% of measurements with automated retinoscopy were within ± 0.5D. 74% of measurement values fell within ± 1D of prescribed values arrived by an optometrist using subjective refraction
	**Amblyogenic risk factors (5)**							
13	Effectiveness of the **GoCheck Kids** Vision Screener in Detecting Amblyopia Risk Factors [12]	Peterseim et al., 2017, Am. J. Ophthalmol	Prospective study	From a working distance of 28 inches, the smartphone app takes photos of children’s eyes in a dark room. Using photorefraction classification was made into “no risk” and ‘risk factors identified” for Amblyopic risk factors (ARF) This was compared with visual acuity, motility, Anterior segment evaluation, cycloplegic retinoscopy, fundus evaluation	206 children	Children aged 6 months to 6 years	Manifest strabismus exceeding 15 PD was excluded	The prevalence of ARF in this study was found to be 36%. In comparison to conventional standard of clinical evaluation of ARF, **GoCheck** demonstrated a sensitivity of 76.0% (95% CI: 64.6%–85.1%) and a specificity of 67.2% (95% CI: 58.4%–75.1%) in identifying ARF. The positive predictive value was 57.0% (95% CI: 50.2%–63.6%), while the negative predictive value stood at 83% (95% CI: 75.3%–88.2%)
14	Positive predictive value and screening performance of **GoCheck Kids** in a primary care university clinic [21]	Law et al., 2020, J AAPOS	Retrospective study	**GoCheckKids** measurements were compared with visual acuity tests, IOP, stereoacuity, ocular alignment and ocular motility measurements were recorded. Anterior segment evaluation, dilated fundus evaluation with cycloplegic (cyclopentolate 1%) retinoscopy	2963 children	Records of children between 3 to 48 months presenting to the UCSF clinic between February 2017 and August 2018 with abnormal **GoCheckKids** results	Beyond age group, with normal results of GoCheckKids	172 had abnormal photo screener result Main reasons for referral were anisometropia and hyperopia (54%), exclusively hyperopia (18%), myopia (8%), anisometropia with myopia (11%) and anisometropia (9%). 16(28%) had astigmatism according to the application criteria Among 57 patients satisfying ARF (Amblyopic Risk Factor) criteria, PPV was 50% (95%CI, 41% to 60%) with lowest values (26%) present among younger age group (3–12 months). High values of PPV were obtained among Hispanic groups (95% CI, 57% −100%, *P* < 0.01)
15	Evaluation of a smartphone photoscreening app to detect refractive amblyopia risk factors in children aged 1–6 years [15]	Arnold et al.,2018, Clin Ophthalmol	Prospective multicenter	Eye metrics like first Purkinje image acquisition, crescent width, pupillary diameter and limbal diameter were used to calculate photorefraction values. The interpretations were: Amblyogenic risk factors identified, no risk factor identified, not gradable or to retake on another date. This was compared to cycloplegic refraction	287 images of children	Children between 1–6 years attending paediatric ophthalmology practices in Alaska, Arizona, California, and Pennsylvania	Exclusions included poor-quality images, a history of ocular surgeries (such as squint correction, congenital cataract, or glaucoma surgery) that impacted photorefraction, and other factors influencing measurement accuracy	The manual test identified 72.83 true positives for amblyogenic risk factors. For astigmatism, there were 12 manual and 16 automated false negatives, while for accommodated hyperopia, the manual and automated false negatives were 11 and 16, respectively. The sensitivity and specificity of the manual grading were 76% and 85%, with a positive predictive value (PPV) of 0.76 and a negative predictive value (NPV) of 0.85. In comparison, the automated grading showed a sensitivity of 65% and specificity of 83%, with PPV and NPV values of 0.69 and 0.80, respectively
16	Vision screening using a smartphone platform [34]	Debert et al., 2022, Rev Paul Pediatr	Retrospective Cross sectional	Photoscreening was done with application in landscape and portrait mode at 0.7 m, this was compared to visual acuity, motility, cover tests, Slit lamp anterior segment evaluation, cycloplegic (cyclopentolate 1%) refraction and fundus evaluation were done	157 children	Children from 5 to 7 years were recruited	Who did not undergo comprehensive eye exam	Seven had amblyogenic risk factors, 67% was the prevalence of ARF determined by conventional exam, five had strabismus (four convergent and one divergent), one had high hypermetropia, and one had myopic astigmatism and anisometropia Sensitivity 84%, specificity 74%, positive predictive value was 86% and 70% was the negative predictive value
17	Smartphone photography for screening amblyogenic conditions in children [28]	Gupta et al 2019, Indian J Ophthalmol	Prospective cross sectional	Smartphone grading of photography of the pupillary red reflexes in comparison to ocular findings and refraction	250 children recruited	First visiting patients ages 5 to 8 years of the eye outpatient department were recruited	Refractive errors less than 3 D and anisometropia less than 2 D	23.6% were diagnosed to have amblyopic risk factors. Fifty-nine were abnormal by standard clinical evaluation. The agreement between Primary Investigator (PI) and the first ophthalmologist was 83.2% and that of PI and 2nd ophthalmologist was 87.1%. The interrater agreement (kappa value) between the two ophthalmologists showed almost perfect agreement of 0.928. Mean sensitivity and specificity of smartphone detection was 94% and 91%
	**Leukocoria Diagnosis (3)**							
18	**EyeScreen**: Development and Potential of a Novel Machine Learning Application to Detect Leukocoria [17]	Bernard et al., 2022, Ophthalmology Science	Technology validation cross sectional	Four gaze photos were taken per participant. In dim light rooms. Image processing followed classification by an ocular oncologist as normal, abnormal or leukocoria. Data was resized, augmented, and reduced to dataset mean. These images were used for training a machine learning model with training and testing sets for both normal and abnormal image sets	4000 photos of 1457 children	Consenting participants of children aged less than 18 years were recruited	Images of poor quality or those deemed ungradable were excluded	In this study, abnormal red reflex indicative of ocular pathology was reported in 222 participants from the training set and 55 individuals from the testing set used to train the algorithm The image testing set, consisting of 291 participants, achieved a sensitivity of 87% and a specificity of 73%. The area under the ROC curve was 0.93, while the area under the precision-recall curve was 0.77
19	Autonomous early detection of eye disease in childhood photographs [5]	Munson et al., 2019, Sci. Adv	Retrospective Cohort study	Manual screening of photographs for the presence of faces was done by 3 researchers. **CRADLE App** was used on photos which were retrospectively collected. Photos of leukocoria were classified as pathological or physiological and not leukocoria or as leukocoria	20 cases and 20 control	For those with ocular disorder birth – 3175 days of age and control group till 2501 days with defined criteria of usable photographs	Both eyes of child were closed	23,248 photos of 20 kids with ocular disorder and 29,734 photos of 20 normal kids were used. 20 children were detected to have retinoblastoma (7), coat’s disease (2), bilateral cataract (1), anisometropic amblyopia (1) and bilateral hypermetropia (1). In 80% of children diagnosed with eye disorders, the application identified leukocoria in photographs an average of 1.3 years (95% CI: 0.4–2.3 years) before clinical diagnosis Sensitivity was 90% [95% CI [76.9–100], specificity was 20% [95% CI (2.5–37.5)], accuracy was 55% [95% CI (39.6–70.40)] and intrinsic true positive (ITP) rates were 31.9% and intrinsic false positive (IFP) rates were 0.7% among non- leukocoria individuals
20	Evaluation of a Free Public Smartphone Application to Detect Leukocoria in High-Risk Children Aged 1 to 6 Years [13]	Vagge et al., 2019, J Pediatr Ophthalmol Strabismus	Prospective single center	The **CRADLE** smartphone app was used to record leukocoria presence in the eye and compared to comprehensive evaluation including cycloplegic refraction with (tropicamide 1% and phenylephrine 2.5%) and fundus examination was done	122 children	Children aged 1 to 6 years who visited the University Eye Clinic of Genova, IRCCS Ospedale Policlinico San Martino, or the Queen Sirikit National Institute of Child Health for eye examinations	Any condition that hindered the capture of a clear and adequate image	Among the 244 cases, nine eyes (3.6%) were diagnosed with amblyogenic cataract, one child (0.4%) had stage 5 retinopathy of prematurity, and three children (1.2%) were found to have retinoblastoma. The app demonstrated a sensitivity of 15.38% (95% CI: 1.92%–45.45%) and a specificity of 100% (95% CI: 98.48%–100.00%). The negative likelihood ratio was 0.85 (95% CI: 0.67–1.07)
	**Stereoacuity Applications (2)**							
21	Screening for Stereopsis of Children Using an Autostereoscopic Smartphone [25]	Yang et al., 2019, J Ophthalmol	Observational	Smartphone was presented in comparison to imitations of Lang Stereotest 1 and 2, pass test 3, Random dot stereo acuity test	51 children	Children aged 3–6 years attending Second Hospital of Jilin University of China	Those who did not fall into the criteria for inclusion were excluded	Lang stereoacuity test I, 47 individuals had stereoacuity levels less than 1200. For the 2nd variant of Lang stereoacuity test, 46 individuals had values less than 600. Forty-two children had values ≤ 480″. Thirty-two individuals had values ≤ 400 with Dinosaur stereoacuity test which were accurately detected by autostereoscopic smartphone and standard tests Wilcoxon signed rank test P values were greater than 0.05. Inter test agreement (kappa) Lang stereotest I was 0.905, Lang stereotest II was 0.875, for Pass test it was 0.916, for the Dinosaur stereoacuity test it was 0.840. Random dot stereo acuity test was 0.852. All the weighted kappa were higher than 0.84
22	Evaluation of stereoacuity with a digital mobile application [16]	Bonfanti et al., 2021, Graefes Arch	Experimental (cross sectional)	Upon passing Lang I test, **Stereoacuity Test (SAT)** application was used with anaglyphic (red green) glasses and compared to TNO, Weiss MKW test. Tilting the reading lantern about 20 degrees placed 30 cm away from the desk surface without back reflection, at a uniform distance of eye from source, with moving slide bars were used to set colours. This application administered a set of images based on a staircase algorithm and the correct angle of disparity was deduced	497 children	Children aged between 6 to 10 years with parental consent	Participants with no stereopsis with Lang stereoacuity test 1 and “absence of one of the tests explained in Stimuli and devices”	Significant difference was found between tests F (2,992) = 143.14 *p* < 0.0001 Mean value of stereoacuity was 69, 57.6, 51.1 for TNO, Weiss MKW, SAT. Limits of agreement after tracing Bland Altman plots for SAT and Weiss test 0.36, between TNO and App was 0.34 Intraclass correlation coefficient between tests show a moderate correlation ICC = 0.532, *p* < 0.0001 between the app and TNO 0.53, *p* < 0.0001 between the app and Weiss 0.49 *p* < 0.0001
	**Retinopathy of Prematurity Test (2)**				)			
23	Smartphone-based fundus imaging for evaluation of Retinopathy of Prematurity in a low-income country: A pilot study [29]	Choudhary et al., 2023, Pak J Med Sc	Cross-sectional validation study	Flash on video mode of smartphone and + 28 D lens (Volk) was used for dilated eyes of babies. It was compared to Indirect Ophthalmoscopy (standard). Photos were taken from the videos and indirect identifiers were used followed by observer rating. Cohen’s kappa statistics was used to calculate the interrater agreeability with indirect ophthalmoscopy	63 images of 43 babies using purposive sampling	Babies with diagnosis of stage of ROP (Stage-0, 1,2,3,4 or plus/pre-plus disease)	All babies who had undergone interventions (intravitreal injection, laser, normal, non-ROP diseases	For detecting plus or pre-plus disease, Cohen's kappa values were 0.84 and 1.0 for Rater-1 and Rater-2, respectively, in comparison with the findings from the gold standard, indirect ophthalmoscopy. There was strong agreement in identifying any stage of ROP, with kappa values of 0.65 and 1.0 for Rater-1 and Rater-2. The inter-rater agreement for plus disease and ROP staging also showed high values, with kappa scores of 0.84 and 0.65. In terms of image quality, Rater-1 classified 3.17% of the images as unacceptable and 96.83% as acceptable, while Rater-2 rated 1.58% as unacceptable and 98.41% as acceptable
24	Smartphone guided wide-field imaging for retinopathy of prematurity in neonatal intensive care unit – a Smart ROP (SROP) initiative [27]	Goyal et al., 2019, Indian J Ophthalmol	Observational study	Condensing lens‑smartphone‑ black tube device assembly (CSD) with + 20 D, + 28D, + 40D lens was used to visualise various parts of the eye, videos of the visualised areas were recorded on smartphone and screen shots were taken. This was preceded by indirect ophthalmoscopy	228 eyes of 114 preterm babies	Between January to May 2018 preterm babies were recruited less than 34 weeks with birth weight less than 1750 g	Eyes with hazy media, poorly dilating pupil, those on obstructing ventilatory support	Total ROP incidence was 23.6%. Type 1 ROP was seen in 20 (8.7%) of the eyes. Smartphone photography was used to diagnose 28 eyes as ROP out of the 54 eyes with ROP
	Applications for strabismus measurement (6)							
25	Validation of **StrabisPIX**, A mobile application for home measurement of ocular alignment [31]	Phanphruk et al., 2019 Translational Vision Science and Technology	Prospective cross sectional	Nine gaze photos taken with the application were compared to clinical photographs by 3 observers. Binocular alignment, vertical and horizontal versions, pupil size, eyelid position and head posture were assessed	30 patients 2–81 years	Participants aged 2 years and above with diagnosis of strabismus were recruited	Those unable to follow instructions, unable to fixate in instructed directions	Intermittent strabismus was present in 12 individuals and 2 people had complex strabismus. Intra-rater agreement was moderate to almost perfect (k 0.49–1.00) which was similar to Inter-rater agreement (k 0.44–1.0). Pupil size abnormality detection showed fair (k 0.23) agreement for intra-rater agreement and was between poor and fair for inter-rater agreement. Other anomalies were detected at similar rates for the ocular parameters; alignment abnormalities were detected at higher rate in the images of StrabisPIX application (89%) compared to 77% for clinical photos. Clinical photos were better for detection of versions, head posture, eyelid position and pupil size
26	Validation of the **2 WIN Corneal Reflexes** App in children [32]	Racano et al., 2021, Graefes Arch	Prospective cross sectional	**2 WIN Refractometer** Corneal Reflexes (CR) App was used. Comparison with alternate cover test with distance-based prism ocular alignment	167 children	Participants aged 2 to 14 years were recruited during their first visit to the Paediatric Ophthalmology and Strabismus Clinic at Rovereto Hospital, between August 2016 and March 2019	30 children were excluded as the application could not recognize, among them, 16 were lesser than 5 years	137 children were diagnosed with strabismus High sensitivity and specificity of the app was 79.2% and 86.2%. PPV and NPV of the app was 86.4%, 78.9%. In younger children, sensitivity was 88.9, specificity was 80%. For esotropia and exotropia, 88.9% and 50% was the sensitivity and 91.6% and 100% were the specificity. For vertical deviations 33.3% sensitivity and 89.3% specificity was seen, however with poor correlation R^2^ value = 0.168, p = 0.001. For esotropia good correlation R^2^ value = 0.582 Wilcoxon signed rank sum test but including the poor correlation seen for exotropia, p value was not statistically significant for both the conditions (p(eso) 0.765, p(exo) 0.056)
27	A mhealth application for automated detection and diagnosis of strabismus [3]	Michelline et al., 2021, Int. J. Med. Inform	Retrospective study	Brightness reflected by light from the smartphone is compared with landmark parameters from the corneal limbus This is compared to the findings of the strabismologist	224 children	patients aged 5–15 years from 2015 to 2017, including deviations up to 90PD	Those with corneal abnormalities, microphthalmia, significant nystagmus, could not read1.0/1.0 on the Snellen chart, with 40″ of arc in Titmus stereoscopic test	Two cutoff points of 6 and 11 prismatic dioptres (PD). were analysed. With the 6PD cut off more (30) patients were detected by the application to have strabismus. Clinician detected *32* children (8.5%) with strabismus. At the 6 PD cutoff, the prevalence of squint was 21.8%, with a fair kappa value of 0.43 (95% CI: 0.38–0.48), which was statistically significant (*p* < 0.0001). The sensitivity was 89.47% (95% CI: 66.8%–98.7%), specificity was 84.39% (95% CI: 78.68%–89.0%), and accuracy was 84.5% (95% CI: 79%–89%). At the 11 PD cutoff, the kappa value was 0.49 (95% CI: 0.35–0.61), also statistically significant (*p* < 0.042)
28	Evaluation of a Hirschberg Test-Based Application for Measuring Ocular Alignment and Detecting Strabismus [14]	Garcia et al., 2021, Current Eye Research	Prospective cross sectional inter -rater agreement study	Alternate prism cover test (APCT) at distance and near fixation. 2 Smartphone photographs of the face were taken 33 cm away from the face for distance and near fixation Centroids of fixation were drawn at the center of the pupil, and pixels between the eyes were measured and converted into metric scale	43 patients	Patients aged 12 to 60 years with a horizontal corneal diameter (HCD) ranging from 10.86 mm to 12.54 mm (mean 11.7 mm ± 0.84) were included. Participants were required to maintain fixation on both distant and near accommodative targets for each eye	Subjects above 60 years, with nystagmus, irregular limbus contour, corneal opacities or alterations with/without eyelid anomalies, non-detection of eye region, corneal reflex	Alternate prism cover test showed that 14 people had strabismus. From 137 photographs, 14 had exotropia at distance fixation and 17 had exotropia at near fixation The sensitivity of detecting horizontal strabismus photographically was 92.86%, and specificity was 7.69%. Specificity of vertical strabismus was 14.81% due to lesser sample size. For image processing of strabismus, specificity was 8%, PPV was 52% for distance and 54.17% for near. For study of strabismus measurement, for horizontal strabismus (distance fixation) bias was 6.125Δ 95% CI [−25.07 and 37.32 SD ± 15.91). Variation of measurement for horizontal strabismus is 4 to 25Δ. Vertical strabismus distance fixation bias is 4.375 D [−6.56 to 15.31]. Bland Altman plots at 95%CI for distance fixation were 21 Δ with coefficient of repeatability 15.241 for horizontal strabismus. For horizontal strabismus there were narrower limits of agreement. (−7.615 to 8.615 Δ)
29	Development and Preliminary Evaluation of a Smartphone App for Measuring Eye Alignment **(EyeTurn)** [8]	Pundlik et al., 2019, Translational Vision Science and Technology	Cross sectional	Phoria measurements taken with the app at near fixation (40 cm) were compared to those obtained using the modified Thorington (MT) method in normally sighted subjects (n = 14). Eye deviations measured by the app were also compared with results from the cover test with prism neutralization (CTPN; n = 66) and the Synoptophore (n = 34) in subjects with strabismus	66 participants aged between 4 to 63 years were recruited	Participants with a prior diagnosis of horizontal strabismus (either constant or intermittent exotropia or esotropia) and no other visual impairments	6 app error, 1 operator error, 1 statistical outlier	MT measurements showed 12 people with exophoria and 2 with esophoria The range between −24 Δ (esodeviation) and + 15 Δ (exodeviation) The mean within-subject differences for MT measurements ranged from −1 Δ to 2.8 Δ, with a 95% confidence interval. Repeated measures ANOVA yielded F(2, 41) = 0.198, p = 0.98. The mean intra-subject measurement difference was 0.4 Δ, with a 95% CI of 2.3 Δ. The app's measurements closely aligned with MT values (linear regression: slope = 0.94; intercept = −1.12; R^2^ = 0.97, p = 0.001), with a root mean squared difference of 1.7 D between the app and MT For CTPN measurements, the strabismus angle varied from ± 60 Δ, with the smallest angle recorded at 6 Δ. Among the participants, 49 had exotropia and 18 had esotropia. CTPN measurements were consistent with the app’s measurements (linear regression: slope = 0.95, intercept = −0.86, *R*^2^ = 0.95, *p* < 0.001). However, angular deviations measured by the app were larger than those from the synoptophore (linear regression: slope = 1.15, intercept = −3.19, *R*^2^ = 0.91, *p* < 0.001)
30	Quantitative measurement of horizontal strabismus with digital photography (**EyeStrab)** [23]	Dericioglu et al., 2019, J.Pediatr. Ophthalmol	Cross sectional, validation study	Photographs of eyes at a known image distance were taken with the developed smartphone app. Measurements were taken of distances from geometrical corneal centre to light reflex (RD) and corneal diameter. RD/CD ratio was calculated of each gaze angle Images of retrospectively recruited strabismus patients were also used to apply the ratio Comparison: Krimsky or prism test by strabismologist	511 photographs of 72 individuals (3–40 year)	First visit to Marmara University Department of Ophthalmology or diagnosed patients visiting Paediatric Ophthalmology and Strabismus Department were recruited	Those with latent strabismus, could not fixate to the camera, or with corneal ocular surface problems	Twenty-four were diagnosed with exotropia and 48 with esotropia There was a high correlation between app measured gaze angle and specialist measured gaze angle (*r* = 0.966 *P* < 0.001) According to Bland Altman analysis, there was no significant difference between estimated and real deviation angle (*p* > 0.05), 0.983 was Cronbach’s a coefficient The estimated deviation and specialist measurement differed at an average of 0.68 ± 6.1 degrees
	Applications for assessment of eyelids and ptosis (2)							
31	Photographic assessment of eyelid position using a simple measurement tool paired with cell phone photography in a paediatric population [20]	Prakalapakorn et al.,2021, J AAPOS	Prospective “proof of concept” study? cross sectional	An ophthalmologist recorded Marginal reflex distance 1 (MRD1) and Palpebral Fissure (PF) height, Levator function(LF) with a mm ruler with nearest 0.5 mm. Laminated mm ruler attached to spectacle frame. Smartphone was used to capture a photo of the same. This was graded by an ophthalmologist and non ophthalmogist	70 participants (1–20 years old)	Those who could fixate on an object, and follow spoken directions	Poor photograph quality, with non-visualization of corneal reflex or no image being available for grading photographs	Ten participants (14%) had strabismus, four (6%) had unilateral ptosis, one (1%) was diagnosed with Pompe disease, and one (1%) had Down’s syndrome. Both graders rated all photographs as good or fair for assessing Interpalpebral Fissure Distance (IPFD) and Marginal Reflex Distance (MRD1), with 70% of the photographs deemed adequate for evaluating levator function (LF). The limits of agreement (in mm) between clinical and photographic assessments were 1.1 (− 1.5 to 3.8) for IPFD, 0.7 (− 1.8 to 3.1) for MRD1, and 1.1 (− 3.5 to 5.7) for LF Intraobserver repeatability during clinical examination was excellent for IPFD (ICC = 0.81, 95% CI: 0.67–0.89), MRD1 (ICC = 0.88, 95% CI: 0.78–0.93), and LF (ICC = 0.94, 95% CI: 0.89–0.97). For the photographic technique, repeatability was fair for IPFD (ICC = 0.44, 95% CI: 0.15–0.65), good for MRD1 (ICC = 0.74, 95% CI: 0.57–0.85), and good for LF (ICC = 0.77, 95% CI: 0.61–0.83). Interobserver assessments of the photographs showed excellent agreement for IPFD (ICC = 0.94, 95% CI: 0.91–0.96), MRD1 (ICC = 0.96, 95% CI: 0.94–0.97), and LF (ICC = 0.92, 95% CI: 0.88–0.94)
32	Parent-provided photographs as an outcome measure for childhood chalazia [18]	Erzurum et al., 2022, April, J. Pediatr.Ophthalmol	Prospective cross-sectional study	4 photos were taken of the eye with chalazion with smartphone photography by the parents of the participants compared with clinical evaluation	60 participants	Children between 7 months to 16.5 years with new or recurrent chalazion ≥ 2 mm diameter on at least one eyelid	Those without chalazion	85 chalazion cases were identified. Sensitivity of 84%, Specificity of 97%, kappa coefficients (95% CI) of 0.72 (0.55–0.88), 0.65 (0.49–0.82), and 0.19 (− 0.03 to 0.42) for depth, horizontal and vertical positioning of chalazion by observer compared to clinical evaluation was noticed
	Applications for assessment of corneal diameter (1)							
33	Comparing the Use of Smartphone and Vernier Calipers for Corneal Diameter Measurement in Nigerian Term Neonates [6]	Mgbafulu et al., 2023, Niger J Clin Pract	cross sectional study	Reading with Smartphone was taken in primary gaze in supine position with the application and a horizontal line was drawn to demarcate the horizontal corneal diameter (CD). This was done from 3 to 9 clock hours with 3 readings being taken. This was compared to manual corneal caliper measurement by 2 observers	1213 term neonates	Neonates within 1 week of birth, with or without ocular disorders with parental consent	Those that did not meet inclusion criteria	The mean corneal diameter (CD) measured using calipers was 10.01 ± 0.29 mm for the right eye (RE) and 10.03 ± 0.24 mm for the left eye (LE). For smartphone-based measurements, the mean CD was 9.98 ± 0.21 mm for the right eye and 10.00 ± 0.29 mm for the left eye, with consistent results from the second investigator. No statistically significant difference was observed between the mean CD values obtained by both instruments and investigators (*p* > 0.05). Intra-user variability, assessed through repeated measures ANOVA, also showed no significant difference between the caliper method (p = 0.904) and smartphone photography for investigator 1 and 2 (*p* = 0.879)

Subgroup or sensitivity analysis could not be applied due to the inclusion of different studies, with different study design, and tools being used and varied outcomes being studied.

### Study quality assessment

Quality of studies was checked using Joanna Briggs Institute Critical Appraisal Checklist for Analytical Cross-Sectional Studies [47]. The checklist consisted of answers such as “yes”, “no”, “unclear” and “not applicable”. The study quality was assessed by the total percentage of yes out of the total number of answered questions. This process was also independently done by two masked reviewers (S.V. and Y.A.). The tabulated study quality assessment has been mentioned in Table 4(added at the end of the document).

**Table 4:** Study quality analysis table based on JBI criteria for studies

			Question								% Yes	Quality (High/moderate(mod)/low)
S.no	Source	Publication date	Q1	Q2	Q3	Q4	Q5	Q6	Q7	Q8		
1.	Zhao et al	06 Jun 2019	Y	Y	Y	Y	U	Y	Y	Y	87.5	High
2.	Rehman et al	10 May 2023	Y	Y	Y	Y	NA	NA	Y	Y	75	High
3.	Chen et al	26 Jan 2023	U	Y	Y	Y	NA	NA	Y	Y	62.5	Moderate
4.	Azis et al	11 Sep 2019	Y	Y	Y	Y	NA	NA	Y	Y	75	High
5.	Han et al	19 Aug 2019	Y	Y	Y	Y	NA	NA	Y	Y	75	High
6.	Raffa et al	07 Jul 2022	Y	Y	Y	Y	NA	NA	Y	Y	75	High
7.	Srivastava et al	11 Oct 2019	Y	Y	Y	Y	NA	NA	Y	Y	75	High
8.	Siti et al	06 Nov 2020	Y	Y	Y	Y	NA	NA	Y	Y	75	High
9.	Chun et al	20 Mar 2020	Y	Y	Y	Y	NA	NA	Y	Y	75	High
10.	Ma et al	13 Jul 2020	Y	Y	Y	Y	NA	NA	Y	Y	75	High
11.	Luo et al	28 Feb 2022	Y	Y	Y	Y	N	Y	Y	Y	75	High
12.	Aggarwal et al	10 Aug 2022	Y	Y	Y	Y	NA	NA	Y	Y	75	High
13.	Peterseim et al	24 Dec 2017	Y	Y	Y	Y	NA	NA	Y	Y	75	High
14.	Law et al	12 Jan 2020	Y	Y	Y	Y	NA	NA	Y	Y	75	High
15.	Arnold et al	23 Aug 2018	Y	Y	Y	Y	NA	NA	Y	Y	75	High
16.	Debert et al	06 Jun 2021	U	Y	Y	Y	NA	NA	Y	Y	62.5	Moderate
17.	Gupta et al	08 May 2019	Y	Y	Y	Y	NA	NA	Y	Y	75	High
18.	Bernard et al	07 Apr 2022	Y	Y	Y	Y	NA	NA	Y	Y	75	High
19.	Munson et al	02 Oct 2019	U	Y	Y	Y	NA	NA	Y	Y	62.5	Moderate
20.	Vagge et al	14 May 2019	Y	Y	Y	Y	NA	NA	Y	Y	75	High
21.	Yang et al	31 Oct 2019	N	Y	Y	Y	NA	NA	Y	Y	62.5	Moderate
22.	Bonfati et al	27 Apr 2021	Y	Y	Y	Y	NA	NA	Y	Y	75	High
23.	Choudhary et al	31 Jan 2023	Y	Y	Y	Y	NA	NA	Y	Y	75	High
24.	Goyal et al	06 Jan 2019	Y	Y	Y	Y	NA	NA	N	N	50	Moderate
25.	Phanphruk et al	29 Mar 2019	Y	Y	Y	Y	NA	NA	Y	Y	75	High
26.	Racano et al	06 Jan 2021	Y	Y	Y	Y	NA	Y	Y	Y	87.5	High
27.	Joana et al	15 Jun 2021	Y	Y	Y	Y	NA	NA	Y	Y	75	High
28.	Garcia et al	19 Apr 2021	Y	Y	Y	Y	NA	NA	Y	Y	75	High
29.	Pundlik et al	08 Feb 2019	Y	Y	Y	Y	NA	Y	Y	Y	87.5	High
30.	Dericioglu et al	23 Jan 2019	Y	Y	Y	Y	NA	NA	Y	Y	75	High
31.	Prakalapakorn et al	14 Oct 2022	Y	Y	Y	Y	NA	NA	Y	Y	75	High
32.	Erzurum et al	18 Mar 2022	Y	Y	Y	Y	NA	NA	Y	Y	75	High
33.	Mgbafulu et al	01 Nov 2023	Y	Y	Y	Y	NA	NA	Y	Y	75	High

## Results

### Search results

**Fig. 1:**
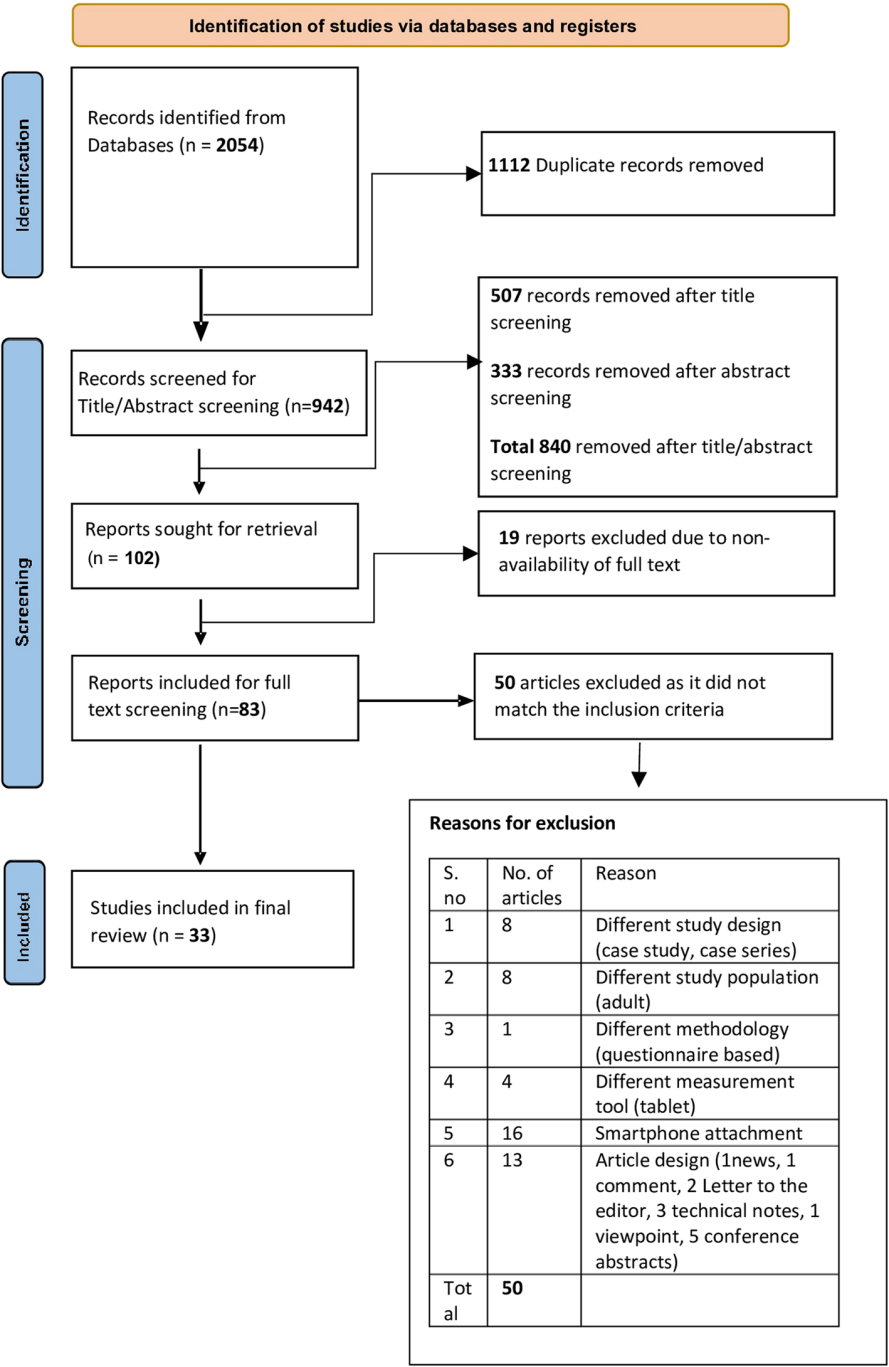
Flow diagram for study selection for systematic review

Totally, 2054 articles were retrieved following database search. After removing 1112 duplicates, 942 articles were considered for title and abstract screening. Following initial screening, 507 records were removed after title screening and 333 were removed following abstract screening following the inclusion and exclusion criteria. Among the 102 articles obtained at this stage, due to non-availability of full text, 19 articles were excluded. The remaining 83 articles were screened and from these, 50 failed to match the inclusion criteria. Thirty-three articles were included the final study. The selection process and the rationale for exclusion after full text screening have been depicted in Fig. 1. The characteristics of each study have been described in detail in Table 3.

### Study design characteristics

Most of the studies were cross sectional (22[66.67%]). There was one (3.03%) clinical trial, one development and validity study, one technological validation cross sectional study, one experimental study, one non interventional randomized cross-sectional study, two observational studies, and four retrospective studies. All the studies elaborated sufficiently on the efficacy of smartphones for detecting paediatric eye diseases.

### Study geographical location characteristics

The studies comprised of smartphone applications being developed in diverse surroundings like United Kingdom [20, 35], Malaysia [9, 33], China [4, 11, 25], Illinois, Tennessee [12, 19], Arizona [15, 21, 34], Michigan [17], California [5, 13], Massachusetts [8, 22, 31] and Florida [18], United States. Germany [10], Slovenia [26], India [24, 27, 28, 30], Pakistan [29], Republic of Korea [7], England [22], Italy [16, 32], Brazil [3], Philippines [14] Turkey [23] and Nigeria [6] were also regions from where studies were chosen.

### Characteristics of study samples

The mean sample size of the studies was 478 ± 891 children**,** ranging from 30 to 3652 participants in one study. The age of the participants was between newborn to adolescents (up to 18 years).

About 16 (48.48%) studies were conducted among a broad age group (newborn up to adolescent age group),15 studies (45.45%) were conducted among the preadolescent age group and 2 studies were conducted among the adolescent age children.

### Effectiveness of smartphone applications

Among the diseases studied using smartphone, 12(36.36%) applications were utilized in the detection of refractive error using visual acuity tests and the principle of photorefraction [4, 7, 9–11, 19, 22, 24, 26, 30, 33, 35], of which eight were named applications and four were unnamed applications.

Five (15.15.%) studies focussed on detecting amblyogenic risk factors [12, 15, 21, 28, 34], six (18.18.%) applications were present for strabismus detection [3, 8, 14, 23, 31, 32], three(9.09%) were used for leukocoria detection [5, 13, 17], stereoacuity level detection was attributed by two [16, 25] (6.06%) applications, one (3.03%) application was reviewed for eyelid position detection [20], one (3.03%) for chalazion detection [18], one(3.03%) smartphone utility was in the detection of corneal diameter [6], and two (6.06%) applications were directed at retinopathy of prematurity (ROP) detection [27, 29].

JBI critical appraisal checklists were used to check the quality. Most of the study quality was high (28(84.85%)) and five moderate quality studies (15.15%) were present.

### Review of visual acuity applications

#### Vision and visual acuity testing applications

Most apps used tumbling E optotypes to administer the test among children at distances of two meter (Peek Acuity, V@home) [10, 35] for distance, six meters distance (Vis Screen app) and few applications used 40 cm as the near visual acuity distance [33]. Siti et al. [9] in their applications used two distance measurements of 1.5 m and three meters.

#### Peek acuity

This app has undergone multiple validation studies. This application could be deployed in school screening and is good in settings which require high reliability and good correlation.

In the study by Rono et al. in 2017 [48] among general population, seventy-five percent of patients were identified as needing referral, with the first and second assessors showing referral agreement rates of 84.8% and 86.4%, respectively, and moderate kappa values of 0.55 and 0.58. The community volunteers using the application demonstrated a sensitivity of 91% (95% CI: 87.7%–93.7%) and a specificity of 78.1% (95% CI: 71.6%–83.6%) for identifying referable eye conditions. The positive predictive value stood at 88.9% (95% CI: 85.3%–91.8%), while the negative predictive value was 81.8% (95% CI: 75.5%–87.1%). Need for age wise bracketed validation studies were recommended.

In another study by Rono et al. in 2021 [49], it was found that healthcare attendance at the primary level improved with usage of the Peek acuity smartphone application, compared to clinical standards. In the intervention group using smartphone, 1429 attended the primary care clinic and 522 attended in the control group with traditional methods of examination (Rate of difference was 906 per 10 000 [95% CI 689–1124; *p *< 0·0001]).

Among studies done in children, Zhao et al. in 2019 [35] compared Peek Acuity application results with the standard evaluation technique, and observed sensitivity ranging from 83 to 86% for detecting decreased vision and 69% to 83% for identifying referable ocular diseases. The highest sensitivity (100%) was found for children aged between three to five years and 39% was the sensitivity to detect abnormal visual acuity.

#### Vis screen application and AAPOS vision screening application

Administered at test distances of three meter and compared with Snellen chart and picture optotype Lea symbol chart, Vis screen [33] reported by Rehman et al. demonstrated a right eye presenting visual acuity sensitivity, specificity, PPV and NPV of 55.6%- 88.4%, 94.7%—99.3%, 57.9%- 81.7% and 96.8%—99% respectively. K value for intra-rater agreement and inter-rater agreement was 0.85 and 0.75 respectively. AAPOS vision screening smartphone application [19] studied by Chen et al. could also be a reliable tool for visual acuity measurement. The area under receiver operating curve (AUC) for the internal validation set and external validation set was 0.940 and 0.843 respectively and by the non-health profession caregivers it was 0.859. This showed great screening potential to be used by varied health background users.

Apollo Infant Sight [11] application utilises cartoon like optotypes to measure visual acuity based on responses by the children. Visionathome [10] application and Smart optometry [26] applications use ETDRS chart optotypes and tumbling E optotypes. The former application uses two meters for distance visual acuity testing and 40 cm for near visual acuity testing. Smart optometry utilises Rosenbaum pocket vision screener for near visual acuity testing and six meters distance visual acuity testing with clear chart 2 version at six meters. Most of the above-mentioned applications showed good sensitivity and showed potential to be used as a screening test.

Among the unnamed applications, Srivastava et al., Ma et al., Chun et al. and Siti et al., [4, 7, 9, 24] used smartphone photography at distances of one meter with target looking six meters away. Testing was also done at 1.5 and three meters respectively. The results obtained were compared to cycloplegic refraction and other standards of evaluation.

Luo [22] used the photography mode and Aggarwal [30] et al. used the video mode of the smartphone to record the pupillary reflexes to detect the refractive error. They compared it with autorefraction and cycloplegic refraction values.

## Amblyogenic risk factors detection

### GoCheckKids

Peterseim et al [12] in their study showed by principle of photorefraction and risk stratification their application could be beneficial in amblyogenic risk factors detection. Following multiple validation studies, the results showed a sensitivity of 76.0% (95% CI: 64.6%–85.1%), a specificity of 67.2% (95% CI: 58.4%–75.1%), a positive predictive value (PPV) of 57.0% (95% CI: 50.2%–63.6%), and a negative predictive value of 83.0% (95% CI: 75.3%–88.2%), hence the application can be useful in screening amblyogenic risk factors.

Law et al. in their study [21] showed that among the 57 patients satisfying ARF (Amblyopic Risk Factor) criteria, positive predictive value (PPV) was 50% (95%CI, 41% −60%) with lowest values (26%) present among younger age group (3–12 months). Hispanic groups were reported with high PPV (95% CI, 57% −100%, *P* < 0.01).

Arnold et al. in 2018 [15] compared GoCheckKids application to standard evaluation techniques. 72.83 were the true positives of amblyogenic risk factors, manually. 12 and 16 were the manual and automated false negative values for astigmatism and 11 and 16 were present for accommodated hyperopia. 76% and 85% were the sensitivity and specificity and 0.76 and 0.85 were positive predictive value (PPV) and negative predictive value (NPV) for manual grading and sensitivity and specificity of automated grading results was 65% and 83%. PPV and NPV were 0.69 and 0.80 for automated grading.

Similarly, in a study by Debert et al [34], The Go Check Kids application demonstrated a sensitivity of 84% and a specificity of 74% when compared to conventional methods, with a positive predictive value of 86% and a negative predictive value of 70%

In a study by Gupta et al [28], based on the pupillary red glow, 23.6% were diagnosed to have amblyopic risk factors. Fifty-nine were considered abnormal by standard clinical evaluation. The agreement between PI and the first ophthalmologist was 83.2% and that of PI and 2nd ophthalmologist was 87.1%. The interrater agreement (kappa value) between the two ophthalmologists showed almost perfect agreement with value of 0.928. Mean sensitivity and specificity of smartphone detection was 94% and 91%.

#### Stereoacuity tests

In this review one named and one unnamed stereoacuity application were looked into. Yang et al. [25]found that usage of smartphone application was comparable to standard clinical tests with Wilcoxon signed rank test p values lesser than 0.05. Inter test agreement (kappa) Lang stereo test I was 0.905, Lang stereo test II was 0.875, for pass test it was 0.916, for the dinosaur stereoacuity test it was 0.840 and for random dot stereo acuity test it was 0.852. All the weighted kappa values were more than 0.84.

Bonfati [16] et al. in their study found that limits of agreement after tracing Bland Altman plots for SAT (Stereoacuity Test) and Weiss test was 0.36 and between TNO and the application it was 0.34. Intraclass correlation coefficient between tests show a moderate correlation ICC = 0.532, *p* was < 0.0001. Between the application and TNO (ICC = 0.53), *p* was < 0.0001 and between the application and Weiss test, ICC was 0.49, *p* < 0.0001.

### Applications for leukocoria diagnosis

#### Eye screen

Bernard et al [17], in their study found that the image testing set, comprising 291 participants, yielded a sensitivity of 87% and a specificity of 73%. The area under the ROC curve for diagnosing leukocoria was 0.93, while the area under the precision-recall curve stood at 0.77.

#### CRADLE application

The application was validated in two studies, in one study by Munson et al [5], sensitivity was 90% [95% ci [76.9–100], specificity was 20% [95% ci (2.5–37.5)], accuracy was 55% [95% ci (39.6–70.40)] and intrinsic true positive (ITP) rates were 31.9% and intrinsic false positive (IFP) rates were 0.7% among non- leukocoria individuals. In another study by Vagge et al [13], the application exhibited a sensitivity of 15.38% (95% CI: 1.92%–45.45%) and a specificity of 100% (95% CI: 98.48%–100.00%). The negative likelihood ratio was 0.85 (95% CI: 0.67–1.07).

#### Applications for detection of retinopathy of prematurity

Two articles described the application usage with low resources and fit the eligibility criteria, hence among the many applications, the focus will be on these two articles.

Choudhary et al. in 2023 [29] found that on comparing smartphone videography with the results of indirect ophthalmoscopy (the standard test) in infants with retinopathy of prematurity (ROP), Cohen's kappa values were 0.84 and 1.0 for rater-1 and rater-2, respectively, in identifying plus or pre-plus disease. The overall agreement for detecting any stage of ROP was also high, with Cohen’s kappa values of 0.65 and 1.0 for raters 1 and 2. For plus disease and the stage of ROP, the agreement remained strong, with kappa values of 0.84 and 0.65. Regarding image quality, the first rater classified 3.17% of images as unacceptable and 96.83% as acceptable, while the second rater rated 1.58% as unacceptable and 98.41% as acceptable.

With a similar setup and videography methodology, Goyal [27]et al, utilising a setup of condensing lens-smartphone was able to detect 23.6% as the incidence among preterm babies.

#### Applications for strabismus detection

In this review, six reports were chosen describing Strabismus detection in paediatric age group.

StrabisPIX application [31] studied by Phanphruk et al. was shown to have good intra-rater agreement being moderate to almost perfect (k 0.49–1.00) which was similar to inter-rater agreement (k 0.44–1.0). Pupil size abnormality detection showed fair (k 0.23) agreement for intra-rater assessment and was poor to fair agreement for inter-rater agreement. Alignment abnormalities were detected at higher rate in the images of StrabisPIX application (89%) compared to 77% for clinical photos.

2 WIN Refractometer Corneal Reflexes (CR) application reported by Racano et al. [32]was able to detect 137 children with strabismus. The application had high sensitivity and specificity of 79.2% and 86.2%. PPV and NPV was 86.4%, 78.9%. In younger children, sensitivity was 88.9%, specificity was 80%. For esotropia and exotropia, 88.9% and 50% was the sensitivity and 91.6% and 100% were the specificity values. For vertical deviations 33.3% sensitivity and 89.3% specificity was seen, however with poor correlation (R2 value = 0.168, *p* = 0.001). For esotropia good correlation (R2 value = 0.582) was seen. On including the poor correlation seen for exotropia, p value was not statistically significant for both the conditions (p(esotropia) 0.765, p(exotropia) 0.056).

Michelline et al [3] described another strabismus detection smartphone technology where light reflected from cornea was compared for presence of squint. This method demonstrated a sensitivity of 89.47% (95% CI: 66.8%–98.7%) and a specificity of 84.39% (95% CI: 78.68%–89.0%), with an overall accuracy of 84.5% (95% CI: 79%–89%). The kappa value was 0.49 (95% CI: 0.35–0.61), which was statistically significant (*p* < 0.042) at the 11 PD cutoff point.

Garcia et al [14] used smartphone photography with principle of alternate prism cover test to detect and additional presence of latent squint. The sensitivity of detecting horizontal strabismus photographically was 92.86%, and specificity was 7.69%. Specificity of vertical strabismus was 14.81% due to lesser sample size. For image processing of strabismus, specificity was 8%, PPV was 52% for distance and 54.17% for near. For study of horizontal strabismus (distance fixation) measurement, bias was 6.125Δ (95% CI −25.07 and 37.32 SD ± 15.91). Variation of measurement for horizontal strabismus was 4 to 25Δ. Vertical strabismus distance fixation bias was 4.375 D [−6.56 to 15.31]. Bland Altman plots at 95%CI for distance fixation were 21 Δ with coefficient of repeatability 15.241 for horizontal strabismus. For horizontal strabismus there were narrower limits of agreement. (−7.615 to 8.615 Δ).

Pundlik et al. [8]in their study compared application derived eye deviation values to cover test with prism neutralization (CTPN) and Synoptophore values in subjects with strabismus. Using repeated measures ANOVA, the results showed F(2, 41) = 0.198, with *p* = 0.98. The mean intra-subject measurement difference was 0.4 Δ, with a 95% confidence interval of 2.3 Δ. Measurements from the application were closely aligned with MT values (linear regression: slope = 0.94; intercept = −1.12; R^2^ = 0.97, *p* = 0.001). The root mean squared difference between the application and MT was 1.7D. The CTPN-measured strabismus angle varied within a range of ± 60 Δ.

The smallest strabismus angle was 6 Δ. There were 49 subjects with exotropia and 18 with esotropia. CTPN measurements agreed with application measurement (linear regression: slope ¼ 0.95, intercept¼_0.86, R2 ¼ 0.95, *P* < 0.001). Angular estimates of the application measured deviation were greater than Synoptophore measurements (linear regression: slope = 1.15, intercept −3.19, R2 = 0.91, *P* < 0.001).

Dericioglu et al. [23] found that smartphone photographs taken with similar methodology of corneal reflex measurement found high correlation between the application measured gaze angle and specialist measured gaze angle (r = 0.966 *P* < 0.001). According to Bland Altman analysis, there was no statistically significant difference between estimated and real deviation angle (*p* > 0.05), 0.983 was Cronbach’s α coefficient. The estimated deviation and specialist measurement differed at an average of 0.68 ± 6.1 degrees.

#### Applications for detection of corneal diameter (CD)

Mgbafulu et al. [6]in their study found that for smartphone derived CD, mean was 9.98 ± 0.21 mm and 10.00 ± 0.29 mm for right and left eye, with similar results for investigator 2. The difference in mean CD for both instruments and for both investigators was not significant (*P* value > 0.05). Intra user variability using repeated ANOVA did not show a statistically significant difference when caliper method (*p* = 0.904) was used and when smartphone photography was done by investigator 1 and 2(*p* = 0.879).

#### Applications for detection of eyelid disorders

Regarding documentation of eyelid position with smartphone, Prakalapakorn et al [20] in their study found that Intra-observer repeatability during clinical examination was excellent for interpalpebral fissure distance (IPFD) with an ICC of 0.81 (95% CI: 0.67–0.89), for Marginal Reflex Distance (MRD1) with an ICC of 0.88 (95% CI: 0.78–0.93), and for levator function (LF) with an ICC of 0.94 (95% CI: 0.89–0.97). In comparison, the repeatability of the photographic technique was fair for IPFD (ICC = 0.44, 95% CI: 0.15–0.65), good for MRD1 (ICC = 0.74, 95% CI: 0.57–0.85), and good for LF (ICC = 0.77, 95% CI: 0.61–0.83).

The interobserver assessment of photographs showed excellent reliability for IPFD (ICC = 0.94, 95% CI: 0.91–0.96), MRD1 (ICC = 0.96, 95% CI: 0.94–0.97), and LF (ICC = 0.92, 95% CI: 0.88–0.94). These results confirm the effectiveness of smartphone-based detection for assessing eyelid position.

In the study by Erzurum et al., [18] chalazia could be documented by parents by photographing their children with smartphones with sensitivity of 84%, specificity of 97%, kappa coefficients (95% CI) of 0.72 (0.55–0.88), 0.65 (0.49–0.82), and 0.19 (− 0.03 to 0.42) for depth, horizontal and vertical positioning of chalazion was found when observer values were compared to clinical evaluation.

## Discussion

This manuscript covered a wide range of diseases that were present in children and could be detected by smartphones. The purpose of the study was to provide a comprehensive overview of all applications of a smartphone currently available, exclusive to their utility in detection of eye diseases in children.

In 2020, it was observed that 131 new applications were available [42]. Few studies have been published in literature regarding applications exclusive to ophthalmology [39, 42, 50–53].

Eyes undergo a series of structural and neurodevelopmental changes in the first year of life for clear images to focus on the retina till maturity [54]. Paediatric eyes are unique compared to adult eye in the aspects that amblyopia, strabismus and vision threatening refractive error can occur if not detected early. Additionally, there is an age sensitive nature of treatment that must be started upon detection. Technologies for detection of paediatric ocular diseases must also be sensitive to matching the limits of normal physiology of ocular development and to rightfully label as a pathology [55].

The difficulties of evaluating a paediatric eye can be approached in two arcs. There are challenges faced due to lack of health education and negative attitude with social stigma regarding conventional diagnostic and treatment approaches of childhood eye disorders. There are barriers to communication, and a need to convey inferences through guardians or parents which can lead to potential communication gaps, and a need for multiple sittings of evaluation. In a few cases, general anesthesia may be required for completion of examination [55].

In amblyopia, there may be significant visual disability in one eye but as it does not affect daily activities the parents may be apprehensive to start treatment. Wearing glasses and dependence on them can be viewed as a physical handicap in few societies. Caregivers might take multiple opinions from health professionals and un-trained individuals which may lead to misguided judgements. Hence visual and educational development is affected by barriers such as family, socio-cultural or economic conditions [55].

Socially accepted technology such as the smartphone can aid in breaking the barriers and help in better access of healthcare. Studies are already being conducted on the utilization of smartphone technology solely for the screening of eye diseases in resource limited settings including rural areas which are then referred to higher centers [39, 52].

This review delves into the application of smartphone technology in diagnosing and detecting ocular morbidities in paediatric age group, which were usually applied in outreach settings where traditional diagnostic tools faced logistical challenges. It also presents a technology which is fast, simple and easy to use in community settings. Their effectiveness is demonstrated in detection of refractive errors [4, 7, 9–11, 19, 22, 24, 26, 30, 33, 35], risk factors for amblyopia [12, 15, 21, 28, 34], retinopathy of prematurity [27, 29] among other diseases. In this review, studies that validated the efficacy of smartphone apps such as Peek Acuity [35], Vis-Screen [33], and other applications that assess visual acuity, strabismus [3, 8, 14, 23, 31, 32], and stereoacuity [16, 25] were included. Most of the studies have reported a high level of sensitivity and specificity in comparison to their standard tests. This suggests that smartphone technology could revolutionize early eye disease detection, particularly in resource-limited areas. However, more age-specific validation studies are needed to improve accuracy of the applications, especially with the advent of artificial intelligence.

We reviewed 12 applications centered on refractive error detection. Underreporting of mild to moderate refractive errors, especially hypermetropia due to lack of cycloplegia in studies [4, 24] were diagnostic limitations. Diseases involving retina and optic nerve, that did not affect the ocular media could not be detected [28]. Low sensitivities and lack of sample training set of the disease meant missed diagnosis [13].

Six applications measuring strabismus were included in our study [3, 8, 14, 23, 31, 32]. It was possible to detect manifest and even latent strabismus. Currently strabismus applications digitally imitate pre-existing tests for stereoacuity and not as an innovation to examination [25]. Micro-strabismus could not be detected [16], poor correlation was found for vertical deviation more than eight prism dioptres [32]. Two-dimensional nature of images also prevented the accurate diagnosis of strabismus [3]. Few studies excluded manifest strabismus greater than 15 prism dioptres which itself could be a risk factor for amblyopia [12].

Few applications detected corneal diameter [6], which is beneficial in documenting detection and progression of paediatric glaucoma. For applications detecting ptosis, studies stressed upon the importance of maintaining standard positions of landmarks including head position. Few studies included a slit lamp head rest like apparatus to satisfy this prerequisite [20]. In the evaluation of chalazion, the study found significant results despite clinical deviations from the normal like lid pigmentation that made diagnosis difficult [18].

The findings of the current review are in line with other reviews that focused upon the utilization of smartphone technology in detection of diseases completely or in part. However, most of the reviews were nonspecific and focused on more than just diagnostic efficacy of smartphone applications. They also discussed studies that involved applications for patient care and treatment. Nagino et al. [39] in their review, included 24 studies out of 71(33.8%) that assessed diagnostic efficacy of m-health applications in the general population (both adult and paediatric). They found most of the applications to deliver satisfactory results, however, some of the applications had statistical differences in comparison to standardized tests. Hussein et al. [52] conducted a review including studies that investigated the efficacy of applications for diagnosis, and disease monitoring of ocular disorders. They observed as well that the tools were effective and reliable. However, they focused mostly on adult disorders such as ARMD, glaucoma, and diabetic retinopathy. Only one of the studies on refraction was done in the paediatric age group. Other reviews [42, 53] focused on other aspects such as whether the software development for patient focused eye care applications was in accordance with the epidemiology of the place or not. Our study in comparison focused solely on studies that described diagnostic accuracy of handheld smartphone technologies detecting paediatric ocular disorders specifically.

The language limits of the review may limit the application and generalizability of the review findings to English speaking populations. The non-inclusion of grey literature may restrict the non-published studies which may have alternate findings and hypotheses. Limiting the year of study may restrict the analysis of the evolution of mHealth applications or earlier models that helped screen for ocular diseases. The lack of representing the need of smartphones, internet access [12, 15], download rates, usage ratings and digital literacy required to access the technology [26] restricts the insights that the review can provide regarding real world applicability and practicality of using mHealth technologies in low-resource settings.

We recommend that future prospective studies focusing on these aspects can fill the lacunae in research. We also recommend that though the review addresses the need to inculcate technology in health screening strategies in children, the findings should be interpreted, considering the constraints described in our review.

This systematic review is novel in the following ways: First and foremost, this review focuses upon studies in which smartphone applications are used specifically for paediatric age groups. Also, the applications used for the detection of those conditions are also enumerated. Their diagnostic accuracy has been described in detail. Most of the applications describe conditions that are potentially vision-threatening and amblyogenic in nature.

Secondly, in this review, studies highlight the fact that most of these applications are piloted in community settings where logistical constraints do not allow traditional ophthalmological equipment to be deployed. Also, most of these applications were tested not only among the normal population but participants with disorders as well. The high degree of sensitivity and specificity achieved in the use of these applications as compared to standard tests, pave way for mass-scale utilization of these applications with increased early detection and treatment for preventable eye disorders, even in low-resource settings.

Thirdly, the findings from this review may be acted upon by health policy makers and programme managers, to introduce these technologies into regular screening programs so that preventable ocular disorders may be detected in the community only, at the first point of contact rather than at the clinician’s office/at the hospital, thereby reducing the burden of secondary-level and tertiary-level healthcare facilities with respect to ophthalmological cases. It is understood that the prevalence of preventable ocular disease would come down as well, if these technologies are integrated into blindness control programs all around the world.

## Conclusion

Our study unveils a wide range of smartphone applications exclusive to detection of eye diseases in children. Comprehensive outlines of mechanisms of applications for detection of specific eye diseases with reliability indices have been provided. The role of data specialists in collaboration with experts in health care in cost effective and socially acceptable software development with age bracketed population is recommended in this study.

Eventually, the inclusion of smartphone technology to which young children have an affinity to, with conventional paediatric ophthalmology evaluation techniques, is expected to be beneficial. Even though smartphone technology cannot replace the human touch, it will be advantageous if updates and developments in smartphone technology could aid in dispensing healthcare to remote areas in its integration with telemedicine and daily ophthalmological practice.

## Data Availability

No datasets were generated or analysed during the current study.

## Abbreviations

AAPOS: American Association for Paediatric Ophthalmology and Strabismus
ANOVA: Analysis of Variance
APCT: Alternate prism cover test
ARF: Amblyopic Risk Factor
ARMD: Age related Macular Degeneration
AUC: Area under the curve
App: Application
CD: Corneal diameter
CI: Confidence interval
CINAHL: Cumulative Index to Nursing and Allied Health Literature
CR: Corneal Reflexes
CRADLE: Computer Assisted Detector of Leukocoria
CS: Cross sectional
CTPN: Cover test with prism neutralization
CVR: Corrected Vision in Right Eye
D: Diopter
EBSCO: Elton B. Stephens Company Information Services
ETDRS: Early Treatment Diabetic Retinopathy Study
HCD: Horizontal corneal diameter
ICC: Intra Class correlation coefficient
IFP: Intrinsic false positives
IOP: Intra Ocular Pressure
IPFD: Inter palpebral fissure distance
ITP: Intrinsic true positives
JBI: Joanna Briggs Institute
JMIR: Journal of Medical Internet Research
K: Kappa
LE: Left eye
LF: Levator Function
LOA: Limits of Agreement
logMAR: Logarithmic Minimum Angle of Resolution
MKW: Maddox Kreuzweise test
MRD: Marginal Reflex Distance
MT: Modified Thorington test
Mesh: Medical subject headings
N: No
NA: Not applicable
NPV: Negative predictive value
PD: Prismatic Diopters
PPV: Positive predictive value
PRISMA: Preferred Reporting Items for Systematic Reviews and Meta-Analyses
PVR: Presenting Vision in Right Eye
RD: Distance from the geometrical center of the cornea to the light reflex
RE: Right eye
ROC: Receiver-operator Characteristics curve
ROP: Retinopathy of prematurity
SAT: Stereoacuity Test
TNO: The Netherlands Organization Stereoacuity Chart
TQWK: Tolerant Quadratic Weighted Kappa
TRR: Test-retest reliability
U: Unclear
VA: Visual Acuity
Y: Yes
